# Amniotic Fluid Proteomics Analysis and In Vitro Validation to Identify Potential Biomarkers of Preterm Birth

**DOI:** 10.1007/s43032-024-01457-3

**Published:** 2024-03-07

**Authors:** Siguo Chen, Weizhou Wang, Guanghong Yan, Mengmei Liu, Min Li, Ping Chen, Qingyan Ma, Jinman Zhang, Ying Tang, Linglin Zhou, Dingyun You

**Affiliations:** 1 Yunnan Provincial Key Laboratory of Public Health and Biosafety & School of Public Health, Kunming Medical University, Kunming, Yunnan 650000 China; 2 Department of Orthopedics, The First Affiliated Hospital of Kunming Medical University, Kunming, 650032 Yunnan China; 3 Department of Medical Genetics, First People’s Hospital of Yunnan Province, Kunming, 650034 Yunnan China; 4 The Electron Microscopy Laboratory, Science and Technology Achievement Incubation Center, Kunming Medical University, Kunming, 650000 Yunnan China; 5 Department of Obstetrics, Gejiu City People’s Hospital, Gejiu, 661000 Yunnan China

**Keywords:** Premature birth, Proteomics, Vitamin K–dependent protein Z, Amniotic fibroblast

## Abstract

This study aimed to investigate the regulation of amniotic fibroblast (AFC) function by vitamin K–dependent protein Z (PROZ) during preterm birth (PTB) and its potential role in adverse pregnancy outcomes. Proteomic samples were collected from amniotic fluid in the second trimester, and AFC were isolated from the amniotic membrane and cultured in vitro. The expression of extracellular and intracellular PROZ in AFC was modulated, and their biological properties and functions were evaluated. Clinical analysis revealed a significant upregulation of PROZ expression in amniotic fluid from preterm pregnant women. In vitro experiments demonstrated that PROZ stimulated AFC migration, enhanced their proliferative capacity, and reduced collagen secretion. Overexpression of PROZ further enhanced cell migration and proliferation, while knockdown of PROZ had the opposite effect. PROZ plays a crucial role in promoting the proliferation and migration of amniotic membrane fibroblasts. Increased PROZ expression in amniotic fluid is associated with the occurrence of PTB. These findings shed light on the potential involvement of PROZ in adverse pregnancy outcomes and provide a basis for further research on its regulatory mechanisms during PTB.

**Supplementary Information:**

The online version contains supplementary material available at 10.1007/s43032-024-01457-3.

## Introduction

Preterm birth (PTB) defined as delivery < 37 weeks of gestation, is considered a multifactorial syndrome and has seriously threatened the life and health of mothers and babies [1]. The latest statistics show that the global incidence of PTB is 10.6% (95% CI, 9.0–12.0%), and approximately 15 million preterm babies are born each year. In addition, the incidence is increasing year by year [2]. PTB is now thought to be a syndrome caused by a variety of mechanisms, including infection and inflammation, uterine placental ischemia and bleeding, uterine hyperdistension, stress, or other immune-mediated processes [3]. However, existing studies on PTB have not shown an obvious causal pathway. Therefore, the precise mechanism is not clear. The interaction of multiple risk factors is thought to lead to a transition from uterine quiescence to preterm labor or rupture of membranes [4].

Prelabor rupture of membranes is one of the most common causes of PTB. During pregnancy, the fetal membrane could prevent mechanical damage/immune rejection/microbial invasion, secrete hormones, and block uterotonin from reaching the uterus [5, 6]. The strength and elasticity of membranes are the main influencing factors of premature rupture [7]. Amniotic fibroblast (AFC) are an important component in maintaining the membrane’s strength and elasticity, and early or excessive maturation of AFC can affect the stability of the membranes [8, 9]. The large amount of collagen secreted by AFC is another key factor that determines the tensile strength of the membranes [10, 11], which leads to the membranes becoming thinner and less elastic, leading to membrane rupture [12].

Protein Z (PROZ) is a 62-kDa vitamin K–dependent single-chain sugar egg consisting of 360 amino acids containing the N-terminal γ-carboxyglutamic acids (Gla) domain needed for efficient secretion. Protein Z-dependent protease inhibitor (ZPI) is a serine protease inhibitor that efficiently inhibits activated factor X when ZPI is in complex with PZ [13], and the role of the PROZ-ZPI complex in regulating pregnancy has been demonstrated. The PROZ expression level in plasma gradually increases in the first trimester of pregnancy and steadily decreases at delivery. At the same time, some studies have reported an association between changes in plasma PROZ concentrations and adverse pregnancy outcomes (recurrent miscarriage, stillbirth, preeclampsia, placental abruption, etc.) [14–16]. However, in existing studies, the regulatory relationship between PROZ and PTB by acting on APC is still unclear.

In vitro studies support the anticoagulant effect of PROZ. Deficiency of PROZ may cause hemostatic imbalance and thrombosis, primarily related to inhibition of activation factors X, XI, and IX [17, 18]. AFC are important components of amniotic membranes and important functional cells to maintain the stability of fetal membranes. Relevant studies have found that AFC have decreased stability at the start of childbirth, and related maturation markers, such as type I collagen and inflammatory factors, are significantly increased [19, 20]. There are no detailed studies of PROZ on AFC function. This study comprehensively explores the effect of PROZ on the characteristics and function of AFC and attempts to explore its role.

## Methods and Materials

### Experimental Design and Statistical Rationale

The objective of this study was to explore key proteins affecting PTB by determining the amniotic fluid proteome of women undergoing amniocentesis in the second trimester. We took a prospective cohort approach and divided the two cohorts into preterm and nonpreterm cohorts (sTable 1). After sample preparation and protein digestion of each sample in the cohort, DIA (data independent acquisition) analysis was performed on the machine separately. The resulting DIA raw file was analyzed by LC‒MS/MS (QE-HFX_DDA mode) to obtain the DDA library database. Then, each sample was analyzed separately by LC‒MS/MS (DIA mode), and the above DDA database was used for qualitative and quantitative analysis. To monitor and evaluate the stability of the system and the reliability of the experimental data, a QC sample was inserted in the sample queue for each interval of a certain number of samples (generally a mixture of all samples), and the data consistency of each QC sample inserted during the entire experiment was evaluated. The quality of QC was mainly evaluated by coefficient of variation (CV), principal component analysis (PCA), and Pearson correlation analysis, and the expression factor (FC) was > 1.5 times (upregulated greater than 1.5 times or lowered less than 0.83 times) and the *P* value was less than 0.05 (*t* test) in the significantly different protein screening. Downregulate the number of proteins.

## Subject Enrollment

All experiments were conducted in accordance with relevant guidelines and regulations. Inclusion criteria: (1) singleton pregnancy; (2) pregnant women who undergo examination during pregnancy and leave amniotic fluid samples; (3) met the diagnostic criteria for PTB (delivery between 28 + 0 and 36 + 6 weeks gestation). Exclusion criteria: (1) serious medical and surgical diseases (gestational diabetes, gestational hypertension, preeclampsia, congenital heart disease, psychiatric disease, etc.); (2) taking antidepressant and other psychotropic drugs; (3) stillbirth, birth defects, miscarriage, lack of information, etc. The baseline of the study subjects was obtained through a questionnaire, and the general epidemiological data of the women at the time of pregnancy examination were collected, including age, ethnicity, time of pregnancy at puncture, mode of delivery, gestational week of delivery, sex of the newborn, etc. (sTable 1). This study follows the principles of the Declaration of Helsinki. A total of 183 participants were enrolled in this study, of whom 47 were premature and 136 had normal deliveries. Amniotic fluid samples from 26 women in the preterm group and the control group were analyzed after propensity score matching. First, HPRP grading was performed using an equal amount of pooled samples, and LC‒MS/MS analysis was performed to obtain the DDA library database. Each sample was then analyzed separately by LC‒MS/MS and qualitatively quantified using the DDA database described above.

## Sample Preparation

A sample of amniotic fluid is taken during paracentesis in pregnant women. Each sample is approximately 5 to 10 mL and is aspirated from the amniotic cavity with a puncture needle before the start of surgery. All samples were immediately stored at − 80 °C for further analysis. The protein concentration of amniotic fluid samples was determined by Bicinchoninic acid (BCA). Filtered sample preparation (FASP) was used for protein digestion. For each group, a mixed sample of equal amounts of protein was extracted from each sample for library generation. The proteins were denatured by incubation with 20 mM dithiothreitol for 5 min at 95 °C, followed by 45 min of alkylation with 55 mM iodoacetamide in the dark. Trypsin (1:50) was added to these samples and incubated overnight at 37 °C.

## Liquid Chromatography‒Mass Spectrometry Analysis

Peptides are separated using the High-Performance Liquid Chromatography Mobile Phase A, Easy-NLC 1200 Ultra Performance Liquid Separation System. Mobile phase A was an aqueous solution containing 0.1% formic acid and 2% acetonitrile; mobile phase B was an aqueous solution containing 0.1% formic acid and 90% acetonitrile. After the peptides were separated by an ultrahigh-performance liquid phase system, they were injected into an NSI ion source for ionization and then analyzed with an Orbitrap Exploris™ 480 mass spectrometer. High-resolution Orbitrap is used to detect and analyze peptide precursors and their secondary fragments. Data acquisition is performed using a DDA program for more accurate results.

## Proteomics Data Analysis

DDA data were imported directly into Spectronaut software (SpectronautTM14.4.200727.47784) to build the spectral library. The database uses the human UniProt download database. The search parameters were set as follows: the enzyme was trypsin, the maximum missed cleavage site was 1, the fixed modification was carbamidomethyl (C), the dynamic modification was set to oxidation (M) and acetyl (protein N-term), and the protein identified by database retrieval passed the set filter parameter FDR < 1%. DIA data were processed using Spectronaut™ software, which is the same database used to build the database. The software parameters are set as follows: retention time prediction type is set to dynamic iRT, interference on MS2 level correction is enabled, cross-run normalization is enabled, and all results must pass the set filter parameter *Q* value cutoff of 0.01 (equivalent to FDR < 1%).

## Amniotic Fibroblast Acquisition and Identification

We obtained the amniotic tissue used to extract AFC from three pregnant women who had given birth at full term through spontaneous delivery. The tissue was quickly separated after the placenta was successfully delivered, as shown in sFigure 1. We then rinsed the tissue with D-Hank solution (Chinese Servicebio) and cut it with sterile scissors. The lower epithelial cells were digested twice at 37 °C by placing amniotic tissue into 0.125% trypsin 0.02% DNase. They were washed vigorously with PBS (China Servicebio) to further remove epithelial cells. The remaining amniotic tissue is then digested with 0.1% collagenase at 37 °C to release fibroblasts from the interstitial tissue. The samples were placed into an ultrafiltration tube, centrifuged at 12,000 g at room temperature for 20 min, and rinsed three times with PBS to stop the digestion of collagenase. Isolated AFC were cultured in DMEM (China Servicebio) containing 10% fetal bovine serum and biantibiotics at 37 °C and 5% CO_2_. When the cell confluency reached more than 80%, the old culture medium was discarded, the cells were passaged, the cells were subcultured by one-pass two or one-pass according to the number of cells, and the cells obtained were identified by immunofluorescence staining and flow cytometry.

## AFC Cultures

AFC cultures were cultured in an incubator at 5% CO_2_ and 37 °C. Cells were passaged every 3 days, and cells were seeded into flasks every 25 cm^2^ culture at 3.75 × 10^5^. The control group was cultured with complete DMEM, and the experimental group medium was treated with PROZ recombinant protein (China Cloud Cloning Technology) at different concentrations because the PROZ concentration in normal maternal plasma was approximately 1.3 (1.36 ± 0.61) µg/mL [21], and the PROZ concentration in the culture medium was set to 0.4 μg/mL (low), 1.2 µg/mL (medium), and 2.0 μg/mL (high).

## Cell Migration

AFC were seeded in 6-well plates at 2 × 10^6^ cells/well and cultured to 80% confluence. The cells were incubated for 3 h in an incubator at 5% CO_2_ and 37 °C, and scratches were made vertically by 200 μL pipette tips at the bottom of the wells with the same force and washed twice with PBS. Pictures were taken every 12 h with a microscope for 48 h, and the residual area of the scratch was calculated with ImageJ with an initial area of 0 h.

## Cell Proliferation

AFC viability was determined by the CCK8 method. The cell suspension was seeded in 96-well plates with 2000 cells per well (100 μL/well). The cells were preincubated in an incubator for 12 h to allow the cells to attach. After incubation for 0, 12, 24, 48, and 72 h, 10 μL of CCK8 assay solution was added to each well, and the OD was detected with a microplate reader.

## Cell Apoptosis

AFC were cultured under stimulation for 24 h according to the experimental group and collected by centrifugation (290 g/5 min). Cells were washed in PBS and then resuspended in 200 μL of binding buffer (1 ×) at a density of 2–5 × 10^5^/mL. Cells were treated according to the instructions of the Invitrogen Annexin V-FITC Apoptosis Assay Kit (Thermo Fisher Scientific USA). Flow cytometry was used for data analysis.

## Western Blot

For western blotting, harvested cells were lysed on ice using RIPA lysis buffer (Beyotime) with protease and phosphatase inhibitors for 30 min. Then, the lysate was centrifuged at 20 °C and 4 g for 10 min. The supernatant was transferred to another tube and quantified using the BCA Protein Assay Kit (Thermo Fisher Scientific USA). Loading buffer was added to the supernatant (Biyuntian, China), and the samples were denatured at 100 °C for 10 min. The total protein extract was subjected to SDS–polyacrylamide gel electrophoresis, transferred to a PVDF membrane, and blocked with 5% BSA in TBST. The primary antibody was incubated overnight at 4 °C, and the secondary antibody was incubated at room temperature for 1 h. Finally, the signal was detected, and an image was taken. The primary antibodies we used were as follows: anticollagen I (1:1000, Thermo Fisher, USA) and anti-vitamin-dependent protein Z (1:1000, Abcam, UK).

## Quantitative Real-Time PCR (qPCR)

qPCR was used to detect the expression of mRNA level–related indicators. TRIzol reagent (Ambion Life Technologies (USA)) was used to isolate total RNA from cells. After calculating the RNA concentration, reverse transcription was performed using a cDNA synthesis kit (Takara, Japan). Samples were added according to the instructions using the prepared cDNA template and PCR kit, followed by qPCR using instruments (CFX Connect™, Bio-Rad, USA). α-SMA primers were designed based on NCBI and PubMed statistics. GAPDH was used as an internal reference.

## Overexpression and Knockdown of PROZ

Lentiviral vector infection was performed at 50 and 100 infection multiplex (MOI) with cell number/mL × MOI = TU/mL. The experimental cells were diluted to 60,000 cells/mL in each growth medium. Each lentiviral vector was dissolved in serum-free RPMI1640 or DMEM at 6.0 × 10^6^/mL and 1.2 × 10^7^/ml. Lentiviral vector infection was performed in a mixture containing equal amounts of cell suspension and lysed lentiviral vectors (final concentrations of 50 MOI and 100 MOI, 30,000 cells/mL). Twenty-four hours after infection, the medium was replaced with fresh medium containing 5% FBS and further incubated for 72 h.

## Placental Immunohistochemical Staining

The placenta was divided into three layers: leaf-like chorion, amniotic membrane, and bottom decidua, and a total of five samples of placenta and umbilical cord section were PROZ-stained. Paraffin sections were dewaxed and hydrated with xylene and gradient alcohol. After that, the sections were heated for antigen retrieval. Endogenous peroxidase was inactivated with 3% H_2_O_2_ after cooling to room temperature. The antigen was blocked with 30% normal goat serum for 4 min at room temperature, and the primary antibody was incubated overnight at 30 °C. Biotinylated secondary antibody (Zhongshan Biotech Co., Ltd., China) was incubated for 45 min at room temperature. For immunohistochemical staining, DAB was used for chromogenic staining, and hematoxylin (Chinese Beyotime) was used for nuclear staining. The results were taken by a microscope (Olympus, Japan). Positive results were quantified by ImageJ software. All experiments were repeated three times in independent settings. The data are displayed as the standard ± mean.

The primary antibody used was as follows: anti-vitamin-dependent protein Z Protein (1:150, Abcam, UK).

### Statistical Analysis

All experimental data were statistically analyzed using GraphPad Prism 9.0 or R4.5 software; ImageJ software was used to quantitatively analyze the scratch area and immunohistochemical results. Only two groups used the independent sample *t* test, and when there were three or more groups, one-way ANOVA was used, and then, the LSD *t* test or Dunnett’s *t* test was used for pair-by-two comparison after it was statistically significant. The test level was bilateral *α* = 0.05.

## Results

### Amniotic Fluid Proteomics Analysis

**Fig. 1:**
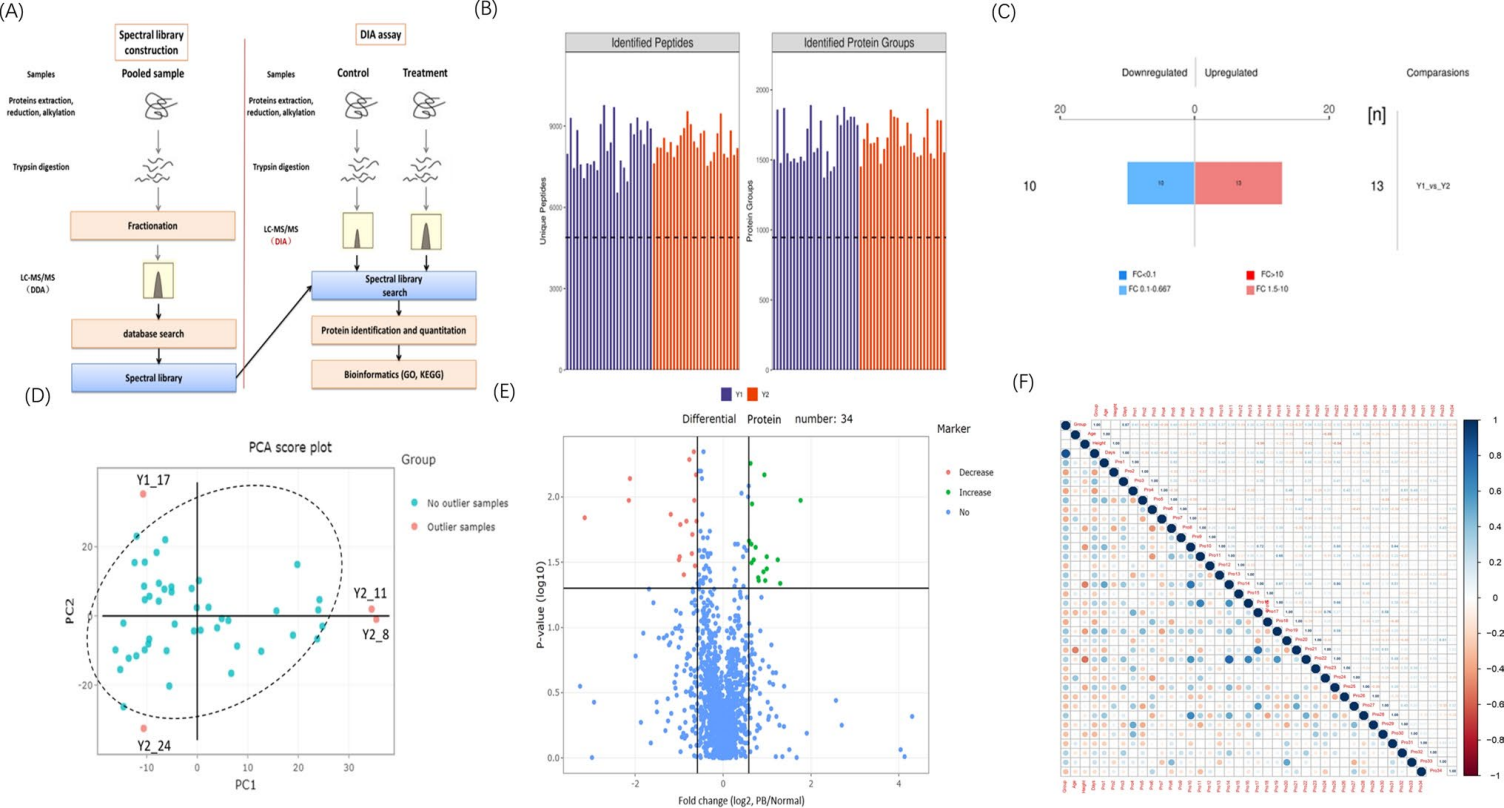
Proteomics analysis of amniotic fluid. **A** DIA quantitative proteomics experimental process. **B** Statistical histogram of DIA identification results; different colors represent different groups. The dashed abscissa line represents the number of proteins at 50% of the total highest identified number.** C** Histogram of protein quantification variance results. **D** Outlier samples, four samples outside 95% of the PCA plot. **E** Volcano map results of single-factor differential analysis, a total of 34 protein performance differences. **F** Spearman rank correlation analysis of 34 differential proteins. Value is correlation coefficient

A total of 183 participants were enrolled in this study, including 136 with normal births and 47 born preterm (sTable 1). We employed the DIA method to analyze the proteome of amniotic fluid samples obtained from 26 preterm births and 26 normal deliveries (control) (Fig. 1A). We compared protein expression differences between the preterm and control groups. In total, 2317 proteins were identified from 14,706 unique peptides (unique peptide ≥ 1). We conducted significant differential protein screening and identified 23 differentially expressed proteins, with 10 upregulated and 13 downregulated (Fig. 1B, C). Additionally, we removed four outlier samples (Fig. 1D) and employed a multivariate partial least squares discriminant analysis (PLS-DA) model to screen for important proteins with variable importance projection (VIP) values greater than 1. This led to the identification of 34 differential proteins (sTable 2), with 17 upregulated and 17 downregulated (Fig. 1E). Furthermore, we performed age correlation analysis on the top 10 differential proteins and found that PROZ exhibited the strongest correlation with days of pregnancy, with a Spearman correlation coefficient of 0.31 (Fig. 1F).

### Amniotic Fluid Proteomic Analysis Showed PROZ as a Differential Protein

**Fig. 2:**
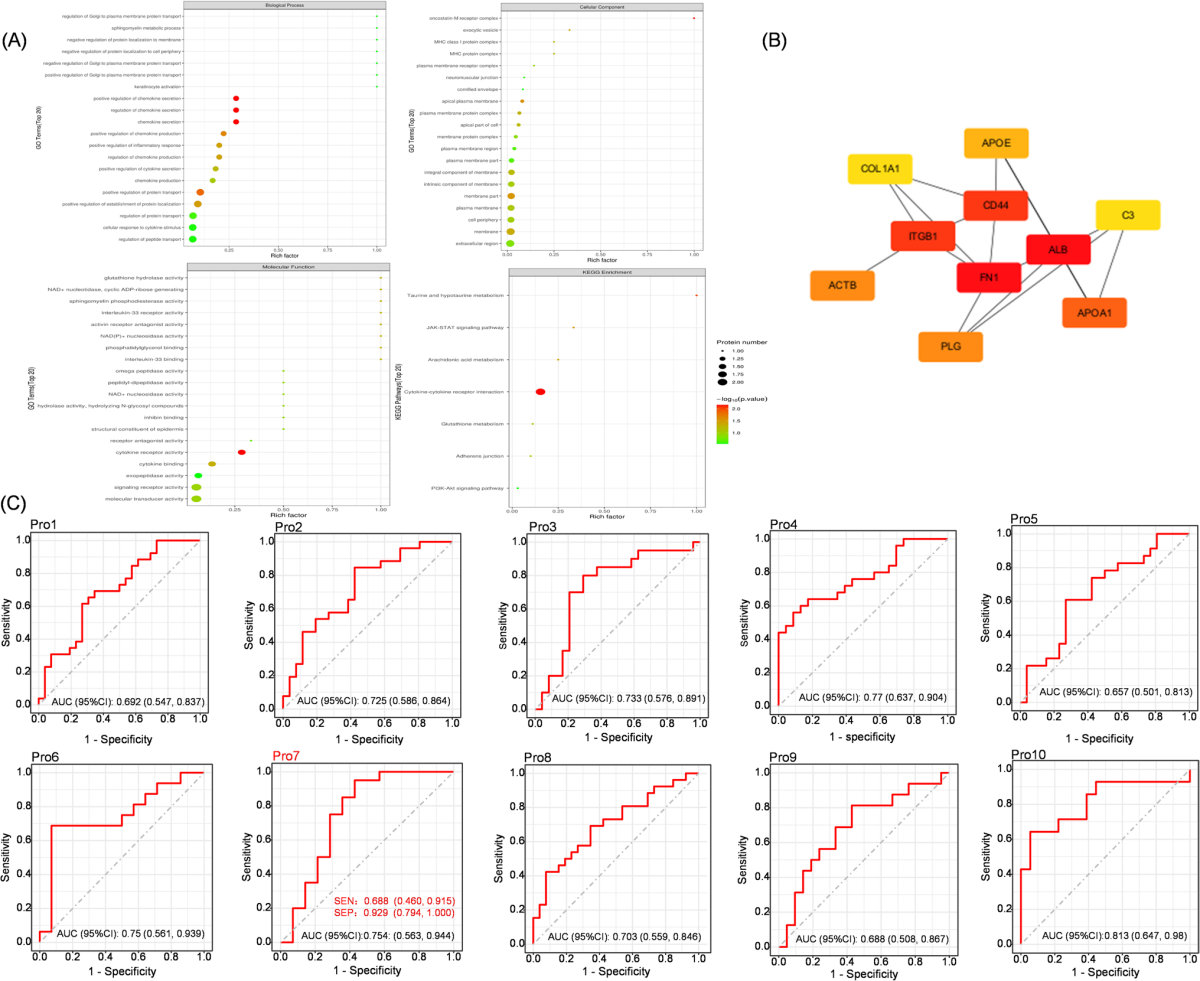
Proteomic analysis of amniotic fluid. **A** Bubble map of GO/KEGG analysis of different potential target genes. **B** PPI network diagram of hub genes among differential potential target genes. **C** ROC curve analysis of the predicted value of PTB in PROZ. The AUC value is the area under the curve. Pro7 is PROZ

We conducted GO and KEGG enrichment analysis on the identified differential proteins (Fig. 2A). Among them, the KEGG pathway enrichment analysis revealed a significant association with cytokine‒cytokine receptor interactions. Subsequently, we constructed a protein‒protein interaction (PPI) network map using the genes corresponding to the differentially expressed proteins and identified hub genes (Fig. 2B). Notably, the occurrence of PTB was influenced by four hub genes. Furthermore, we performed analysis to assess the predictive value of PTB using receiver operating characteristic (ROC) curves. Interestingly, PROZ ranked third in terms of predictive value, with an area under the curve (AUC) of 0.754, sensitivity (SEN) of 0.688, and specificity (SEP) of 0.929 (Fig. 2C). The comprehensive biological information obtained strongly suggests that the upregulation of PROZ expression holds the greatest research value in relation to PTB.

### Successful Extraction of AFC from Human Amniotic Tissue

**Fig. 3:**
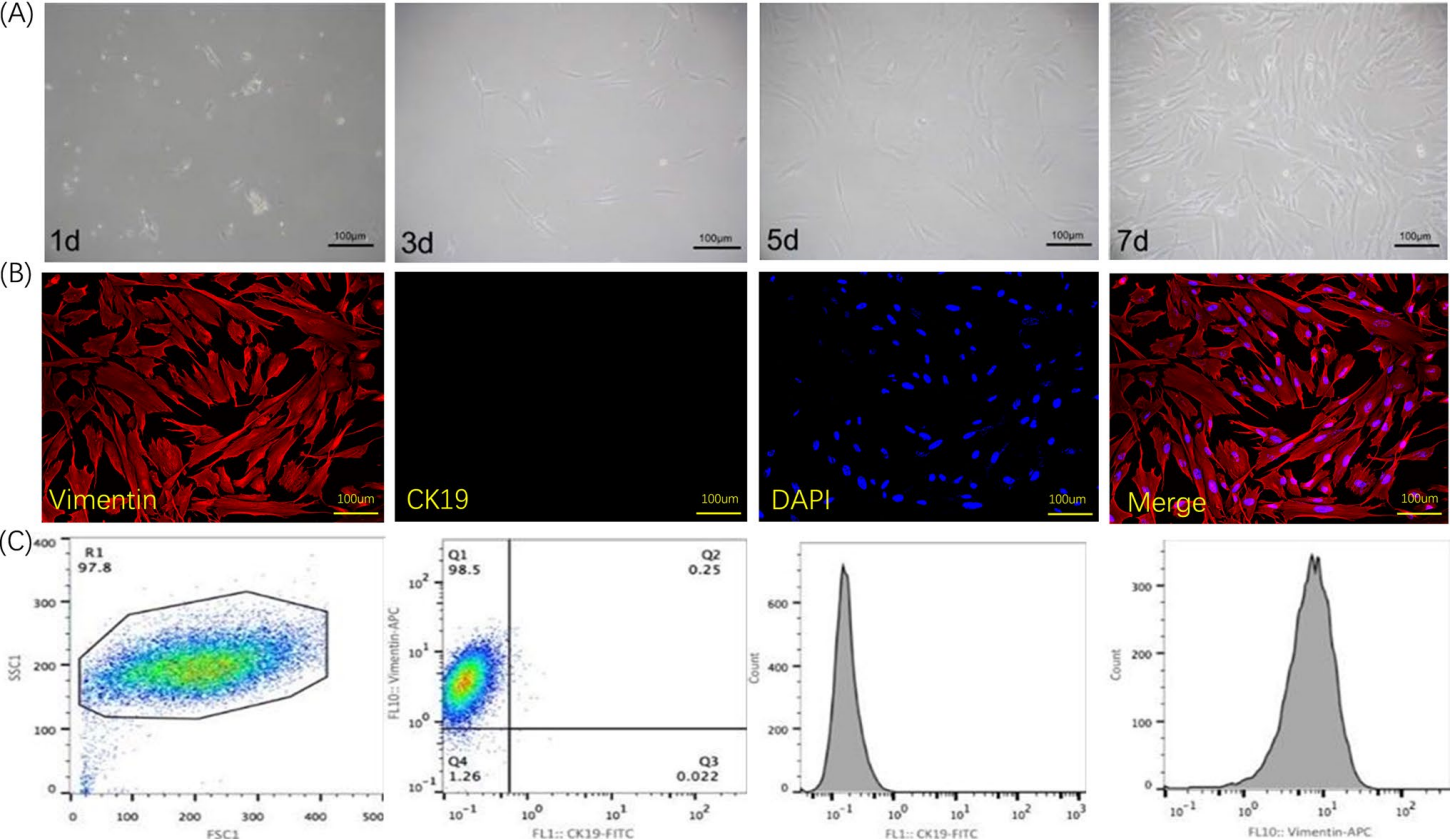
Extraction of AFC. **A** Amniotic membrane acquisition and in vitro culture of fibroblasts. **B** Immunofluorescence identification of AFC. **C** Identification of extracted AFC by flow cytometry. R1 indicates positive expression of vimentin, and Q1 indicates negative expression of CK19

Human amniotic tissue was isolated from the placenta, and primary AFC were extracted (sFigure 1). The AFC were cultured in vitro and exhibited flat, spindle-like morphotypes (Fig. 3A, B). Flow cytometry analysis using specific molecular markers vimentin and CK19 pairs revealed that 98% of the cells expressed vimentin but were negative for CK19 (Fig. 3C).

### The Biological Properties and Functions of AFC Are Regulated by PROZ In Vitro

**Fig. 4:**
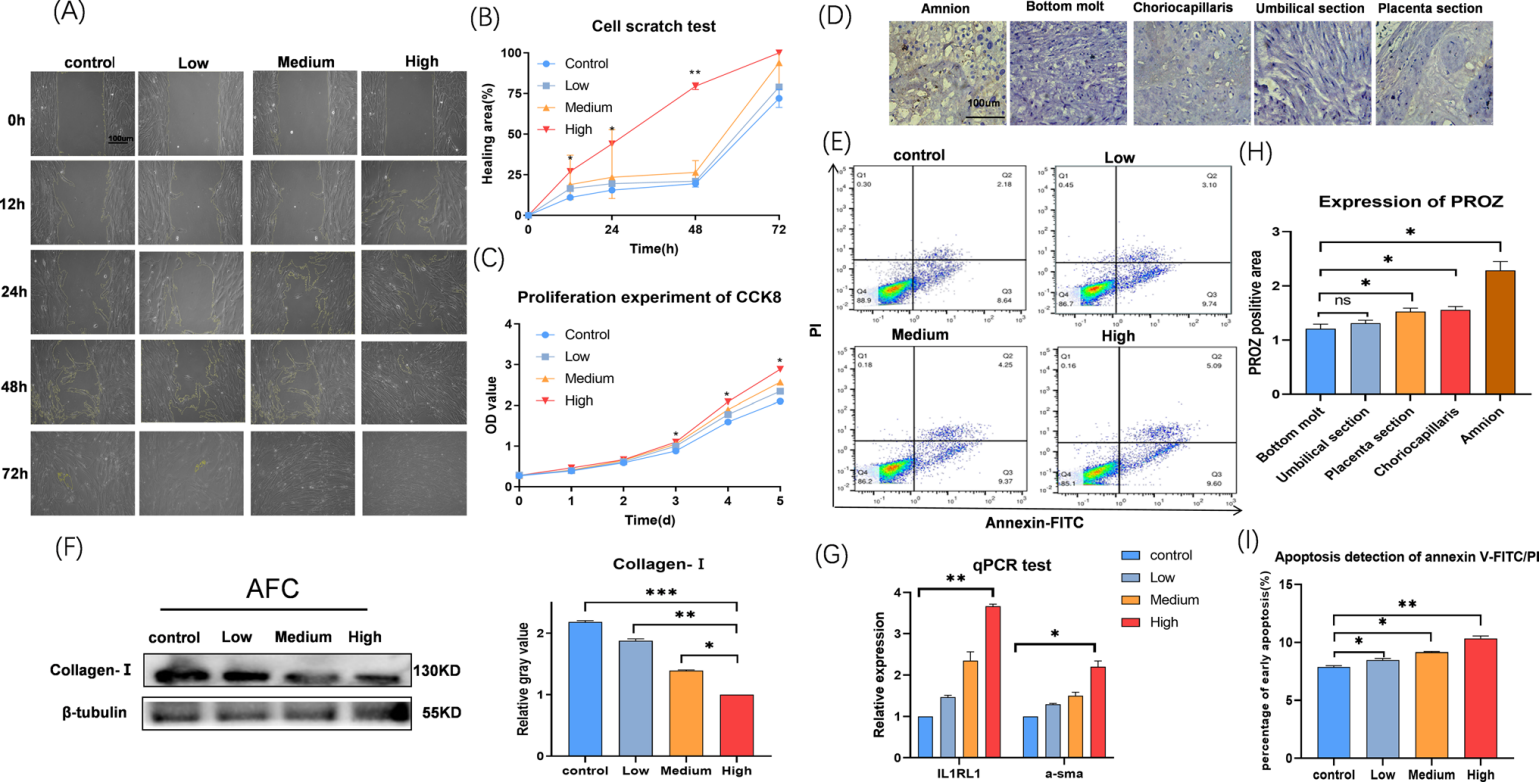
Effect of PROZ on AFC properties. **A** Migration experiment of AFC. The cell scratch test results were photographed with a light microscope. **B** Calculate the area of uncured scratches with an image analyzer. **C **AFC viability assay. The viability of different groups was tested with CCK8, and the results were detected with a spectrophotometer. Vertical axis, absorbance; horizontal axis, time. **D** Immunohistochemical staining of PROZ in each layer of the placenta section. **E** Apoptosis detection by flow cytometry after staining with Annexin V-FITC/PI. Q2 represents advanced apoptotic cells, Q3 represents early apoptotic cells, and Q4 represents living cells. **F** Western blotting was used to detect the expression of collagen I after external intervention with PROZ. **G** qPCR detected IL1RL1 and α-SMA in AFC. **H** Calculate the optical density of each slice by an image analyzer. Vertical axis, optical density value; horizontal axis, layers of the placenta. **I** Analyze streaming data by software; vertical axis, early apoptotic cell percentage; horizontal axis, group. The data are displayed as a standard ± mean. (*P < 0.05; **P < 0.01; ***P < 0.001)

The scratch test results demonstrated that treatment with PROZ enhanced AFC migration ability and accelerated the speed of scratch healing (Fig. 4A, B). Immunohistochemical staining of placental samples revealed that PROZ was primarily distributed in the amniotic layer of the placenta (Fig. 4D, H). The CCK8 assay and flow cytometry analysis indicated that PROZ had a significant impact on fibroblast proliferation and apoptosis (Fig. 4C, E, I). Western blotting results showed that PROZ significantly influenced the expression of collagen I in AFC (Fig. 4F). Additionally, qPCR results demonstrated that PROZ had a significant effect on the expression of intra AFC IR1RL1 and α-SMA (Fig. 4G).

### The Biological Properties and Functions of AFC Are Internally Regulated by PROZ

**Fig. 5:**
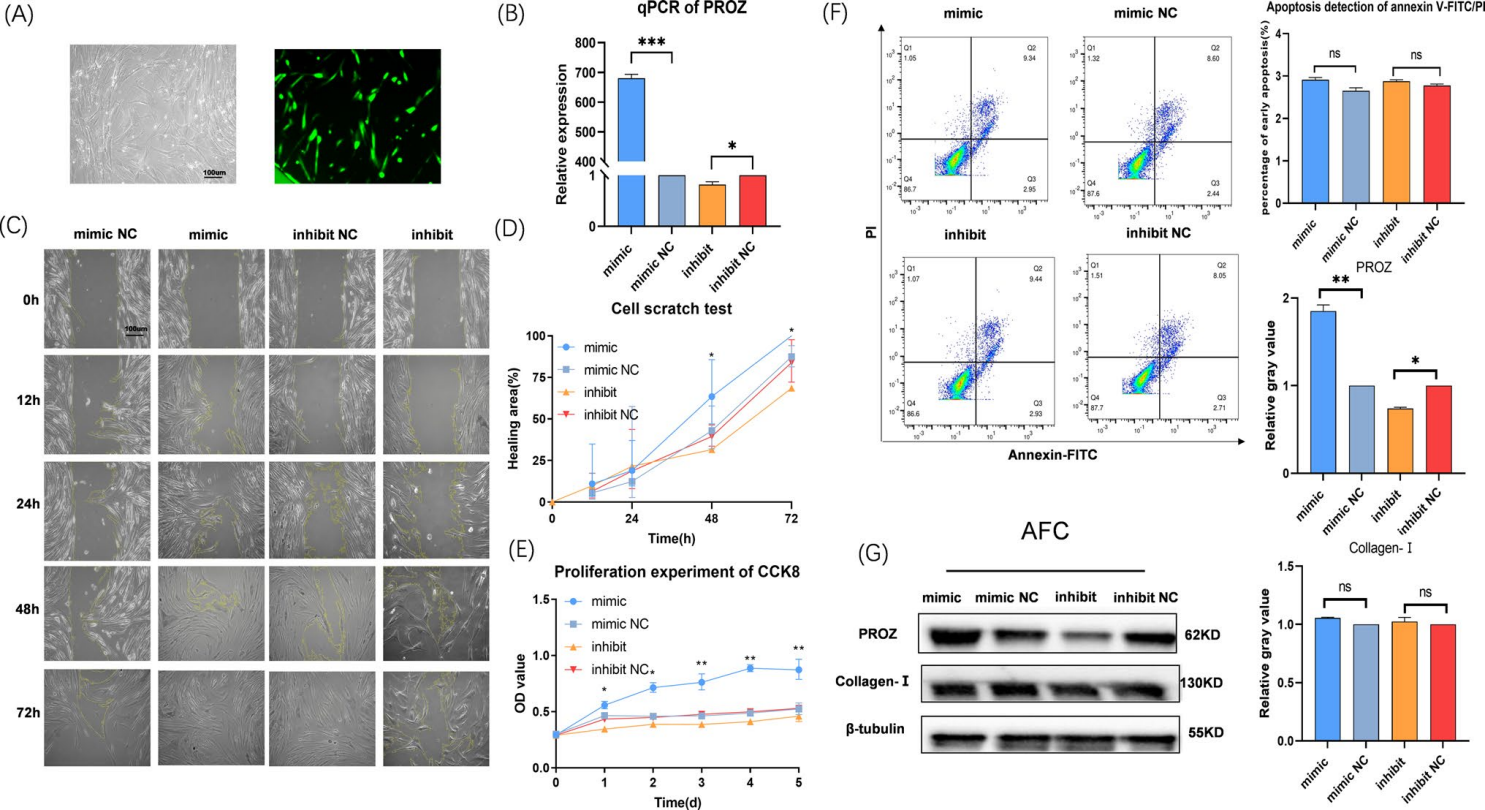
Effect of PROZ mimic, inhibit, mimic NC, and inhibit NC on AFC properties after transfection. **A** Transfection observed under fluorescence microscopy; transfected cells are labeled with green fluorescence. **B** qPCR was used to detect the expression levels of PROZ. **C** AFC migration experiment. The cell scratch test results were photographed. **D** A light microscope was used to calculate the area of uncured scratches with an image analyzer. **E** AFC viability assay after transfection. The viability of different groups was tested with CCK8, and the results were detected with a spectrophotometer. Vertical axis, absorbance; horizontal axis, time. **F** Apoptosis detection by Annexin V-FITC/PI-stained flow cytometry. Q2 represents advanced apoptotic cells, Q3 represents early apoptotic cells, and Q4 represents living cells. **G **Western blotting was used to detect the expression of mimic NC, mimic, inhibit NC, and inhibit PROZ and collagen I. The data provided above are representative figures. All experiments were repeated three times in independent settings. The data are displayed as the standard ± mean. (*P < 0.05; **P < 0.01; *** *P *< 0.001)

The qPCR results demonstrated a significant increase in the relative expression of PROZ in the PROZ mimic AFC and a decrease in the relative expression of PROZ in PROZ inhibitor cells (Fig. 5A, B). Scratch and CCK8 assay results indicated that PROZ mimic AFC exhibited enhanced migration and proliferation capacity, while PROZ inhibitor cells showed weakened migration and proliferation, with no significant effect on apoptosis after transfection treatment (Fig. 5C–F). Western blotting results revealed that the relative expression of PROZ was significantly increased in PROZ mimic AFC and decreased in PROZ inhibitor cells, and had no effect on collagen I expression following PROZ intervention (Fig. 5G).

## Discussion

In previous studies, PROZ has been widely reported to cause adverse pregnancy outcomes [22–24]. However, the majority of studies only reported in vivo phenomena such as a cofactor for the inhibition of factor Xa by ZPI, while others investigated the regulatory role of PROZ in the hemostatic system and proposed possible mechanisms [17, 25]. To date, there have not been any comprehensive studies on how PROZ affects AFC. This is one of the reasons why we decided to focus on PROZ. Additionally, after analyzing the proteins in the amniotic fluid samples of 52 women and using a statistical model to screen for important proteins, PROZ was found to be among the top 10 out of 34 differential proteins. Furthermore, our analysis showed that PROZ had the strongest correlation with the number of days of pregnancy, and when we used the ROC curve to predict preterm birth, PROZ ranked third in terms of predictive value. In cell function experiments, we found that PROZ had the most significant effect on AFC. In this study, we confirmed that PROZ can accelerate amniotic maturation by altering the biological function of AFC.

PTB is the leading cause of perinatal neonatal death, in which spontaneous PTB accounts for approximately 75%. Spontaneous PTB is the focus of PTB prevention [26]. In our proteomic analysis of second-trimester amniotic fluid samples from women with and without PTB, we found that PROZ expression differed significantly between preterm and nonpreterm women in amniotic fluid in the second trimester, and PROZ expression was higher in amniotic fluid from the sample of preterm pregnant women. Based on the role of PROZ in previous studies on adverse pregnancy outcomes [27], it is suggested that excessive expression of PROZ in amniotic fluid in the second trimester may be a precursor to the development of PTB.

Normally, rupture of the amniotic hydranium occurs before labor begins, but in the case of premature rupture of membranes, the amniotic hydranium ruptures before 37 weeks of gestation. This can lead to amniotic fluid leakage, increasing the risk of fetal membrane infection and premature birth. AFC are an important component in maintaining the strength and elasticity of fetal membranes [28], and the early or excessive maturation of AFC can affect the stability of membranes. The functional assay of AFC after stimulation with PROZ illustrated that the PROZ concentration is positively associated with AFC proliferation and migration. Moreover, the trend was similar in PROZ-overexpressing or PROZ-knockdown stable amnioblasts. These changes in biological function indicate that PROZ has the effect of promoting AFC maturation, and we found that AFC after PROZ intervention are more prone to early apoptosis, which is also concentration-dependent, relating to the overmaturity of AFC.

A key factor affecting the strength of the fetal membranes is the large amount of collagen in them, and the weakening of the tensile strength makes the fetal membranes thinner, leading to rupture of the membranes [29, 30]. Our experimental results showed that the secretion of type I collagen in AFC treated with different concentrations of PROZ in cell experiments changed significantly, which was manifested as the higher the intervention concentration was, the lower the expression amount. Studies have reported that the collagen level of the prelabor rupture group was significantly lower than that in the group without premature rupture of membranes. Collagen in the connective tissue of human amniotic tissue is mainly composed of type I and type III collagen [31]. When membrane rupture occurs, type I collagen reduction is most prominent [32, 33]. The main reason for the decrease in type I collagen is the gradual hydrolysis of AFC as they mature, which induces premature rupture of membranes and leads to PTB.

In addition, we also found that the proliferation and migration trend of AFC after lentiviral intervention was the same as that of lentiviral intervention alone, but there was no significant change in apoptosis and collagen secretion, suggesting that there may be a link between collagen secretion and early apoptosis of AFC.

PROZ regulates apoptosis in AFC, and collagen I secretion receptors are located on the surface of the cell membrane. Activation of fibroblasts is closely related to the expression of α-SMA [34, 35]. IL-1β and IL-6 are known to drive inflammation/matrix interactions, including fibroblast activation and dysregulated collagen synthesis [36, 37]. An increase in IL-1β abundance in human amniotic membranes may stimulate ER-phagy-mediated degradation of COL2A3 and COL7A134 proteins in AFC, thereby participating in membrane rupture during delivery [19]. IL-1R is a cytokine receptor that binds to IL-1α or IL-1β, and IL1RL1 is a member of the IL-1R family [38, 39]. Combined with the overexpression of IL1RL1 in amniotic fluid samples from the preterm group in the results of proteomic analysis, we detected the expression of α-SMA and IL1RL1 after PROZ intervention by PCR. As shown in the results, the expression of IL1RL1 in AFC after PROZ intervention was significantly upregulated. Based on this, we speculate that the influence of PROZ on the expression of collagen I may be related to this.

The above results indicate that PROZ can bind to AFC surface receptors, leading to a series of subsequent reactions. However, specific relevant mechanisms still need to be studied in depth.

In conclusion, this study investigated the regulatory role of PROZ in AFC function during the development of PTB. The expression of PROZ was analyzed in amniotic fluid samples, and a significant increase in PROZ levels was observed in amniotic fluid from preterm pregnant women. In cell experiments, PROZ was found to enhance the migration and proliferation of AFC, thereby promoting the maturation of amniotic fluid cells. These findings suggest that PROZ may play a crucial role in promoting premature rupture of membranes and PTB by affecting the secretion of type I collagen through its interaction with surface receptors.

## Conclusion

PROZ enhances the proliferation and migration of AFC and reduces the secretion of collagen I in response to external stimuli. The elevated expression of PROZ in the amniotic fluid of pregnant women is associated with the occurrence of PTB, suggesting that the impact of PROZ on AFC may serve as a key factor in the pathogenesis of PTB.

### Supplementary Information

Below is the link to the electronic supplementary material.

Supplementary file1 (DOC 1947 KB)

Supplementary file2 (DOC 52 KB)

Supplementary file3 (DOC 67 KB)

## Data Availability

The data that support the findings of this study are available from the corresponding author upon reasonable request.

## Abbreviations

PROZ: Vitamin K–dependent protein Z
AFC: Amniotic fibroblast
PTB: Preterm birth

## Declarations

### Animal Studies

No animal studies were carried out by the authors for this article.
