# Supplementary material for: Amniotic Fluid Proteomics Analysis and In Vitro Validation to Identify Potential Biomarkers of Preterm Birth
Source: Reprod Sci. 2024 Mar 7;31(7):2032–42. doi: 10.1007/s43032-024-01457-3 (PMC11217130; doi:10.1007/s43032-024-01457-3)
Supplement: Supplementary file 1 — Supplementary file1 (DOC 1947 KB) [file 43032_2024_1457_MOESM1_ESM.doc]

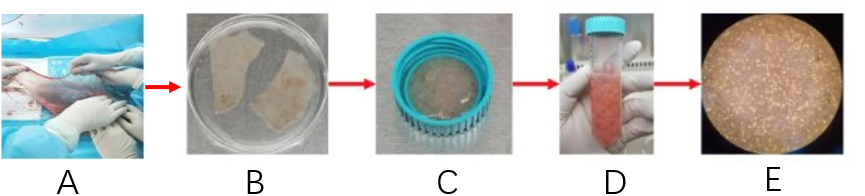


**sFig 1** Amniotic fibroblast extraction process A:Obtain placental amniotic membranes. B: Amniotic membranes are obtained under sterile conditions. C: Cut and shred . D: Wash, digest and filter .E: Primary AFC were obtained by culture.
