# Supplementary material for: Amniotic Fluid Proteomics Analysis and In Vitro Validation to Identify Potential Biomarkers of Preterm Birth
Source: Reprod Sci. 2024 Mar 7;31(7):2032–42. doi: 10.1007/s43032-024-01457-3 (PMC11217130; doi:10.1007/s43032-024-01457-3)
Supplement: Supplementary file 2 — Supplementary file2 (DOC 52 KB) [file 43032_2024_1457_MOESM2_ESM.doc]

**sTable1** Demographic information on pregnant women

| character | All  n = 183 | Preterm birth  n = 47 | Non-preterm birth  n = 136 | *P* value |
| --- | --- | --- | --- | --- |
| **Age** |  |  |  |  |
| Mean (SD) | 32.17 (5.48) | 32.19 (5.46) | 32.16 (5.50) | 0.974 |
| Median (IQR) | 32.00 [27.00;36.00] | 32.00 [27.50;36.00] | 32.00 [27.00;36.00] | 0.962 |
| **Ethnic group** |  |  |  | 0.674 |
| Han Chinese | 146 (79.78%) | 36 (76.60%) | 110 (80.88%) |  |
| minority | 37 (20.22%) | 11 (23.40%) | 26 (19.12%) |  |
| **Pregnancy time** |  |  |  |  |
| Mean (SD) | 140.02 (12.09) | 141.96 (15.59) | 139.35 (10.60) | 0.292 |
| Median (IQR) | 137.00 [131.00;146.50] | 135.00 [130.50;153.00] | 137.00 [132.00;144.00] | 0.75 |
| **Mode of delivery** |  |  |  | 0.396 |
| Cesarean | 103 (56.59%) | 29 (63.04%) | 74 (54.41%) |  |
| Birth | 79 (43.41%) | 17 (36.96%) | 62 (45.59%) |  |
| **Gestational age** |  |  |  |  |
| Mean (SD) | 37.83 (2.45) | 34.45 (2.34) | 38.98 (0.97) | <0.001 |
| Median (IQR) | 38.50 [36.50;39.30] | 35.00 [34.42;36.00] | 39.00 [38.20;39.50] | <0.001 |
| **Neonatal sex** |  |  |  | 0.313 |
| man | 93 (51.67%) | 24 (52.17%) | 69 (51.49%) |  |
| woman | 86 (47.78%) | 21 (45.65%) | 65 (48.51%) |  |
| women and men | 1 (0.56%) | 1 (2.17%) | 0 (0.00%) |  |
