# Supplementary material for: Amniotic Fluid Proteomics Analysis and In Vitro Validation to Identify Potential Biomarkers of Preterm Birth
Source: Reprod Sci. 2024 Mar 7;31(7):2032–42. doi: 10.1007/s43032-024-01457-3 (PMC11217130; doi:10.1007/s43032-024-01457-3)
Supplement: Supplementary file 3 — Supplementary file3 (DOC 67 KB) [file 43032_2024_1457_MOESM3_ESM.doc]

**sTable2** 34 differential proteins screened

| Label | Protein name | **Preterm group median ^a^** | **Control group median ^a^** | **P value** | **Multiplier ratio ^b^** | **VIP** |
| --- | --- | --- | --- | --- | --- | --- |
| Pro1 | Apolipoprotein E isoform 1 (Fragment) | 2.75×10^-05^ | 4.36×10^-05^ | 0.005 | 0.630 | 2.676 |
| Pro2 | Interleukin-1 receptor-like 1 | 4.70×10^-05^ | 3.05×10^-05^ | 0.006 | 1.541 | 2.615 |
| Pro3 | Ig superfamily receptor LNIR | 8.27×10^-06^ | 3.59×10^-05^ | 0.007 | 0.230 | 2.602 |
| Pro4 | IGL c1722_light_IGKV1-5_IGKJ2 (Fragment) | 1.02×10^-05^ | 5.32×10^-06^ | 0.007 | 1.918 | 2.598 |
| Pro5 | cDNA FLJ54854, highly similar to Junctional adhesion molecule A | 1.66×10^-05^ | 2.83×10^-05^ | 0.005 | 0.587 | 2.526 |
| Pro6 | IG c1300_heavy_IGHV3-15_IGHD3-3_IGHJ6 (Fragment) | 3.22×10^-05^ | 2.14×10^-05^ | 0.022 | 1.505 | 2.415 |
| Pro7 | Vitamin K-dependent protein Z variant 1 | 7.44×10^-06^ | 2.19×10^-06^ | 0.011 | 3.395 | 2.321 |
| Pro8 | cDNA FLJ36533 fis, clone TRACH2004428, highly similar to Lactotransferrin (Fragment) | 4.57×10^-04^ | 7.19×10^-04^ | 0.011 | 0.635 | 2.295 |
| Pro9 | Plectin | 1.52×10^-05^ | 3.46×10^-05^ | 0.014 | 0.438 | 2.276 |
| Pro10 | Annexin A13 | 3.65×10^-06^ | 1.61×10^-05^ | 0.011 | 0.226 | 2.260 |
| Pro11 | GLOBIN domain-containing protein | 1.46×10^-04^ | 2.87×10^-04^ | 0.016 | 0.509 | 2.246 |
| Pro12 | IG c1039_light_IGKV4-1_IGKJ2 (Fragment) | 1.15×10^-04^ | 6.85×10^-05^ | 0.024 | 1.671 | 2.232 |
| Pro13 | Oncostatin-M-specific receptor subunit beta | 1.22×10^-05^ | 1.08×10^-04^ | 0.014 | 0.113 | 2.226 |
| Pro14 | Angiotensin-converting enzyme | 1.20×10^-05^ | 1.95×10^-05^ | 0.019 | 0.616 | 2.192 |
| Pro15 | IG c617_light_IGKV3-15_IGKJ5 (Fragment) | 1.17×10^-04^ | 7.47×10^-05^ | 0.023 | 1.561 | 2.153 |
| Pro16 | Mutant hemoglobin alpha 2 globin chain | 7.98×10^-05^ | 1.58×10^-04^ | 0.029 | 0.504 | 2.132 |
| Pro17 | IG c1488_heavy_IGHV4-61_IGHD4-23_IGHJ4 (Fragment) | 7.40×10^-05^ | 3.13×10^-05^ | 0.030 | 2.367 | 2.119 |
| Pro18 | IG c1285_heavy_IGHV3-23_IGHD3-3_IGHJ4 | 9.05×10^-05^ | 5.73×10^-05^ | 0.011 | 1.578 | 2.118 |
| Pro19 | Periostin isoform thy6 | 6.92×10^-05^ | 1.06×10^-04^ | 0.007 | 0.653 | 2.091 |
| Pro20 | IG c925_light_IGKV4-1_IGKJ2 (Fragment) | 7.89×10^-04^ | 1.29×10^-03^ | 0.027 | 0.611 | 2.086 |
| Pro21 | IGH + IGL c591_heavy_IGHV1-69_IGHD3-16_IGHJ6 | 1.03×10^-04^ | 5.16×10^-05^ | 0.029 | 1.990 | 2.077 |
| Pro22 | Fatty acid-binding protein, liver | 1.03×10^-05^ | 2.07×10^-05^ | 0.030 | 0.498 | 2.049 |
| Pro23 | Cystatin-SA | 1.62×10^-05^ | 9.22×10^-06^ | 0.044 | 1.754 | 2.017 |
| Pro24 | IGL c404_light_IGKV3-20_IGKJ1 (Fragment) | 2.43×10^-05^ | 1.22×10^-05^ | 0.036 | 1.991 | 2.014 |
| Pro25 | Chordin-like protein 2 | 2.43×10^-06^ | 4.34×10^-06^ | 0.015 | 0.561 | 2.006 |
| Pro26 | IG c657_heavy_IGHV3-7_IGHD5-12_IGHJ4 | 4.11×10^-03^ | 2.62×10^-03^ | 0.032 | 1.568 | 1.957 |
| Pro27 | cDNA, FLJ94534, highly similar to Homo sapiens capping protein (actin filament) | 2.35×10^-05^ | 1.45×10^-05^ | 0.030 | 1.621 | 1.947 |
| Pro28 | Carcinoembryonic antigen-related cell adhesion | 1.86×10^-05^ | 2.83×10^-05^ | 0.015 | 0.658 | 1.946 |
| Pro29 | Myosin, heavy polypeptide 9, non-muscle | 3.29×10^-05^ | 1.74×10^-05^ | 0.037 | 1.890 | 1.933 |
| Pro30 | IGL c2700_light_IGKV1-5_IGKJ1 (Fragment) | 2.87×10^-05^ | 1.65×10^-05^ | 0.041 | 1.740 | 1.925 |
| Pro31 | IG c224_light_IGKV4-1_IGKJ1 (Fragment) | 2.06×10^-05^ | 1.06×10^-05^ | 0.044 | 1.944 | 1.922 |
| Pro32 | IGL c826_light_IGKV1-9_IGKJ4 (Fragment) | 1.91×10^-05^ | 2.98×10^-05^ | 0.034 | 0.642 | 1.881 |
| Pro33 | c23_heavy_IGHV3-49_IGHD5-24_IGHJ6 | 5.26×10^-06^ | 9.76×10^-06^ | 0.039 | 0.538 | 1.868 |
| Pro34 | IG c1710_light_IGKV3-11_IGKJ4 (Fragment) | 1.47×10^-04^ | 5.96×10^-05^ | 0.046 | 2.462 | 1.615 |

**Not**e: a indicates the result after data cleaning and standardization transformation; b median ratio: preterm/control group
